# Combining Multi-Scale Wavelet Entropy and Kernelized Classification for Bearing Multi-Fault Diagnosis

**DOI:** 10.3390/e21020152

**Published:** 2019-02-05

**Authors:** Nibaldo Rodriguez, Pablo Alvarez, Lida Barba, Guillermo Cabrera-Guerrero

**Affiliations:** 1 Escuela de Ingeniería Informática, Pontificia Universidad Católica de Valparaíso, 2374631 Valparaíso, Chile; 2 Facultad de Ingeniería, Universidad Nacional de Chimborazo, 060102 Riobamba, Ecuador

**Keywords:** stationary wavelet transform, multi-scale entropy, Kernel Extreme Learning Machine

## Abstract

Discriminative feature extraction and rolling element bearing failure diagnostics are very important to ensure the reliability of rotating machines. Therefore, in this paper, we propose multi-scale wavelet Shannon entropy as a discriminative fault feature to improve the diagnosis accuracy of bearing fault under variable work conditions. To compute the multi-scale wavelet entropy, we consider integrating stationary wavelet packet transform with both dispersion (SWPDE) and permutation (SWPPE) entropies. The multi-scale entropy features extracted by our proposed methods are then passed on to the kernel extreme learning machine (KELM) classifier to diagnose bearing failure types with different severities. In the end, both the SWPDE–KELM and the SWPPE–KELM methods are evaluated on two bearing vibration signal databases. We compare these two feature extraction methods to a recently proposed method called stationary wavelet packet singular value entropy (SWPSVE). Based on our results, we can say that the diagnosis accuracy obtained by the SWPDE–KELM method is slightly better than the SWPPE–KELM method and they both significantly outperform the SWPSVE–KELM method.

## 1. Introduction

Early diagnosis of failures of bearings is a key factor to improve both safety and reliability of rotating machinery, intensively used in industrial environments. During the last years, several vibration signal analysis methods has been used to achieve early bearing fault diagnosis. Among them, we can find the empirical mode decomposition (EMD) [1], the local mode decomposition (LMD) [2,3] and the wavelet transform (WT) [4]. While the EMD method can self-adaptively decompose a signal into some intrinsic mode functions (IMFs) based on the local characteristic time scale of the signal [5], the LMD method also self-adaptively decomposes a signal into a series of product functions (PFs), each of which is exactly a mono-component signal [2]. Unlike the EMD and LMD, the WT decomposes a signal into several scales using a wavelet base function. One notable feature of this function is that it can show features of hidden failures [6,7,8,9]. Based on these time-frequency methods for signal decomposition, different entropy features have been used such as Wiener-Shannon’s entropy [10,11], energy entropy [12,13], wavelet energy entropy [14], samples entropy [15], multiscale entropy [16,17], permutation entropy (PE) [18,19,20,21], multi-scale permutation entropy [22,23], generalized composite multiscale permutation entropy [24], multi-scale fuzzy entropy [25], composite multi-scale fuzzy entropy [26], dispersion entropy (DE) [27], multiscale dispersion entropy [28], and improved multiscale dispersion entropy [29]. These entropy features are, in turn, passed on to classifiers such as artificial neural networks (ANN) [3,30,31,32] or support vector machines (SVM) [12,17,18,24,26,33,34].

In particular, authors in [12] used IMFs’ energy entropy to determine whether a failure exists or not. In case of failure, a vector of singular values is passed on to an SVM in order to determine the type of failure. In order to determine whether there is a failure or not, the authors in [18] proposed a hybrid model based on permutation entropy (PE). In case a failure actually exists, the PE of a subset of selected IMFs is calculated and used as the input of an SVM. The SVM will, then, classify the type and severeity of the failure. Yongbo Li [35] investigated the LMD method combined with an improved multi-scale fuzzy entropy. They also used the SVM for the fault diagnosis of the rolling bearings. Authors in [26] studied composite multiscale fuzzy entropy (CMFE) to extract the hidden nonlinear features from vibration signals and then the CMFE features were used as the input of an ensemble SVM to improve rolling bearing fault diagnosis. As we can see, both SVM and ANN are commonly used for the classification of different types of failures in rotatory machines. Unfortunately, during training stages, these methods are quite time consuming, which makes them not very efficient.

To overcome the weaknesses of the vector support machine and the neural network, Huang et al. [36,37,38] proposed a new learning algorithm called extreme learning machine (ELM), which aims to improve tuning time in single-hidden layer feed-forward neural networks. Since then, many researchers have adopted ELM in their works mainly because of its efficiency. For instance, authors in [3] combined LMD and ELM and singular value decomposition (SVD) for bearing failure diagnosis. Here, SV are obtained from the product function matrix are passed on to the ELM as its input. The authors in [3] also demonstrated that LMD–SVD–ELM models performed better than EMD–SVD–ELM models. Authors in [32], proposed an ELM model that is combined with a real-valued gravitational search algorithm. They proposed to use the ensambled EMD method as their classifier. Here, energy features, time–frequency features and SV features are computed using the the ensambled EMD method obtaining very good results when applied on bearing fault diagnosis. In a previous work [39], we proposed an ELM classifier based on a combination of stationary wavelet transform (SWT) and SVD. The SWT is used to separate the vibration signals into a series of wavelet component signals. Then, the obtained wavelet component matrix is decomposed by means of a SVD method to obtain a set of wavelet singular values. Finally, the wavelet singular values are used as input to the ELM for classification among ten different bearing failure types. More recently, in [40] we modified the strategy proposed in [39] by replacing the ELM classifier by the Kernel–ELM (KELM) classifier. Including the KELM classifier led us to better results compared to the ELM classifier. This is mainly because the KELM classifier includes two extra features called the wavelet singular value entropy and the Shannon entropy of the raw vibration signal.

Based on our previous results [39,40], including extensions of the Shannon entropy seems to be an efficient strategy to improve the accuracy of the bearing fault diagnosis. Thus, in this article, we consider integrating stationary wavelet packet (SWP) transform with both dispersion (SWPDE) and permutation (SWPPE) entropies. The SWP transform is an extension of the wavelet transform [41,42,43,44,45]. It has a more flexible decomposition capacity in time and frequency, especially in the high frequency region, and also it is able to distinguish sudden changes in the bearing vibration signal. After the entropy features extraction, the KELM classifier is used to perform automatic fault diagnosis. The KELM classifier is created by replacing the ELM’s hidden activation function with a Gaussian kernel function and so improves the generalisation performance of ELM and reduces time consumption for determining the number of hidden layer nodes [36,37,38]. We choose to use KELM as it has been shown to be very efficient in both classification accuracy and tuning time [37]. Using the proposed extraction methods we can create discriminative fault features by calculating the entropy value (either DE or PE) of each wavelet sub-band signal obtained from the raw vibration signal. Furthermore, discriminative fault features obtained using the proposed methods are more effective than the ones obtained by using both the multi-scale DE [28,29] and multi-scale PE itself [22,23].

We apply our diagnosis methods on two bearing vibration signal databases under variable work conditions obtained in [46,47]. Using these datasets, a comparison among the accuracy obtained by our methods and results obtained by the stationary wavelet packet singular value entropy (SWPSVE)–KELM in [40] is performed.

This work is organized as follows; in Section 2 we present a short description of stationary wavelet packet transform and three different measurements of Shannon entropy. In Section 3 we describe the bearing multi-fault diagnosis algorithm implemented in this paper and the setup we consider for our experiments. In Section 4, we analyse the results obtained by our algorithms. We draw some conclusions in Section 5.

## 2. Wavelet Analysis and Entropy Measures

In this section we briefly introduce stationary wavelet packet transform (SWPT) and three Shannon entropy measures, namely SWPDE, SWPPE, and SWPSVE.

### 2.1. Stationary Wavelet Packet Transform

The SWPT is similar to both the stationary wavelet transform [41,42,43] and discrete wavelet transform (DWT) [44,45]. At the first level of wavelet decomposition, an input signal $\left\{x\left(n\right)=w_{0,0}\left(n\right),n=1,\ldots ,N\right\}$ is convolved with a low-pass filter $h_{1}$ defined by a sequence $h_{1}\left(n\right)$ of length *r* and a high-pass filter $g_{1}$ defined by a sequence $g_{1}\left(n\right)$ of length *r*. Both, the approximation coefficient $w_{1,1}$ and the detail coefficient $w_{1,2}$ are obtained as follows:

(1a) $$\begin{array}{cc} w_{1,1}\left(n\right) & =\sum_{k=0}^{r-1}h_{1}\left(k\right)w_{0,0}\left(n-k\right) \end{array}$$

(1b) $$\begin{array}{cc} w_{1,2}\left(n\right) & =\sum_{k=0}^{r-1}g_{1}\left(k\right)w_{0,0}\left(n-k\right). \end{array}$$

Since no sub-sampling is performed, the obtained sub-band signals $w_{1,1}\left(n\right)$ and $w_{1,2}\left(n\right)$ have the same number of elements as the input signal $w_{0,0}\left(n\right)$. Filters $h_{j}$ and $g_{j}$ are computed by using an operator called dyadic up-sampling. Using this operator, zero values are inserted between each pair of elements in the filter that are adjacent. Thus, the SWPT is defined by the pair of filters (low- and high-pass filters) that is chosen and the number of decomposition steps *J*. For this paper, a pair of Db2 wavelet filters has been chosen [44,47]. In the literature, wave filters with order greater than two have also been proposed [14]. Although, wave filters with order greater than two have better discriminatory potential both in time and frequency domains [14], we found that increasing the order of the wave filters does not lead to better diagnosis accuracy levels. Thus, we chose to use the simplest mother wavelet filter, i.e., Db2.

The general process of the SWPT is continued recursively for j=2,…,J as follows:

(2a) $$\begin{array}{cc} w_{j,2i-1}\left(n\right) & =\sum_{k=0}^{r-1}h_{j}\left(k\right)w_{j-1,i}\left(n-k\right) \end{array}$$

(2b) $$\begin{array}{cc} w_{j,2i}\left(n\right) & =\sum_{k=0}^{r-1}g_{j}\left(k\right)w_{j-1,i}\left(n-k\right), \end{array}$$

where the *i* value denotes the *i*-th sub-band at the (j−1)-th level and the number of sub-bands at the (j−1)-th level is equal to $i=1,\ldots ,2^{j-1}$.

### 2.2. Stationary Wavelet Packet Dispersion Entropy

Let w(n) be a signal corresponding to one of the $D=2^{J}$ wavelet sub-band components, then its stationary wavelet packet dispersion entropy (SWPDE) is calculated through the following steps [48]:

*Step* *1:* The wavelet sub-band signal {w(n)} is normalized between 0 and 1 using the normal cumulative distribution function as follows:
  (3) $$y\left(n\right)=\frac{1}{\sigma \sqrt{2\pi }}\int_{-\infty }^{w(n)}exp\left[\frac{-{\left(t-\mu \right)}^{2}}{2\sigma^{2}}\right]dt,$$
  where μ and σ are the mean and standard deviation of the raw vibration signal of *N* data points.
*Step* *2:* The normalized signal y(n) is mapped into *c* classes with integer indices from 1 to *c* using the following equation:
  (4) $$z^{c}\left(n\right)=round\left(c\cdot y(n)+0.5\right)\;n=1,2,\ldots ,N,$$
  where round(·) denotes the rounding operation.
*Step* *3:* Create multiples *m*-dimensional vector $z_{i}^{c,m}$ as follows:
  (5) $$z_{i}^{c,m}=\left[z^{c}\left(i\right),z^{c}\left(i+1\right),\cdots ,z^{c}\left(i+m-1\right)\right],\;i=1,2,\ldots ,N-m+1.$$
*Step* *4:* Each embedding vector $z_{i}^{c,m}$ is mapped into a dispersion pattern $\pi_{v_{0},v_{1},\cdots ,v_{m-1}}$, where $z^{c}\left(i\right)=v_{0},z^{c}\left(i+1\right)=v_{1},\cdots ,z^{c}(i+\left(m-1\right)=v_{m-1}$. Thus, the number of possible dispersion patterns is equal to $c^{m}$.
*Step* *5:* Calculate the probability of occurrence for each permutation pattern $\pi_{v_{0},v_{1},\cdots ,v_{m-1}}$ as follows:
  (6) $$p\left(\pi_{v_{0},v_{1},\cdots ,v_{m-1}}\right)=\frac{\mathrm{Number}\left\{i|i=1,2,\cdots ,N-m+1;z_{i}^{c,m}\;\mathrm{has}\mathrm{type}\;\pi_{v_{0},v_{1},\cdots ,v_{m-1}}\right\}}{N-m+1},$$
  where N−m+1 denotes the total of embedding vectors.
*Step* *6:* Calculate the normalized SWPDE of the *i*-th wavelet sub-band signal w(n) using Equation (7):
  (7) $$SWPDE\left[w\left(n\right)\right]=\frac{-1}{log\left(c^{m}\right)}\sum_{\pi =1}^{c^{m}}p\left(\pi_{v_{0},v_{1},\cdots ,v_{m-1}}\right)log\left(p(\pi_{v_{0},v_{1},\cdots ,v_{m-1}})\right).$$
  Here, for all the experimental examples, the embedding dimension is set to m=2 and the number of classes is in the range $c=\left[5,8\right]$ [27,29,48,49].

### 2.3. Stationary Wavelet Packet Permutation Entropy

The stationary wavelet packet permutation entropy (SWPPE) of a wavelet sub-band signal $\left\{w\left(n\right)=w_{i}\left(n\right),i=2^{J},n=1,\cdots ,N\right\}$ obtained by using Equations (1) and (2) is calculated through the following steps [50]:

*Step* *1:* Create a set of *m*-dimensional vectors $W_{i}^{m}$ as follows:
  (8) $$W_{i}^{m}=\left[w\left(i\right),w\left(i+1\right),\cdots ,w\left(i+m-1\right)\right],\;i=1,2,\ldots ,N-m+1,$$
  where *m* is the embedding dimension of the vector $W_{i}^{m}$.
*Step* *2:* Each vector $W_{i}^{m}$ is sorted in ascending order with permutation pattern π as follows:
  (9a) $$\begin{array}{cc} W_{i}^{m} & =[w\left(i+j_{1}-1\right)\leq w\left(i+j_{2}-1\right),\leq \cdots \leq ,w\left(i+j_{m}-1\right)] \end{array}$$
  (9b) $$\begin{array}{cc} \pi & =[j_{1},j_{2},\cdots ,j_{m}], \end{array}$$
  where each vector $W_{i}^{m}$ in *m*-dimensional space can be mapped to one of the m! ordinal patters π.
*Step* *3:* Calculate the probability of occurrence for each permutation pattern π as follows:
  (10) $$p\left(\pi \right)=\frac{\mathrm{Number}\left\{i|i=1,2,\cdots ,N-m+1;W_{i}^{m}\;\mathrm{has}\mathrm{type}\;\pi \right\}}{N-m+1},$$
  where N−m+1 denotes the total of embedding vectors.
*Step* *4:* Calculate the normalized SWPPE of the *i*-th wavelet sub-band signal w(n) using Equation (11):
  (11) $$SWPPE\left[w\left(n\right)\right]=\frac{-1}{log\left(m!\right)}\sum_{j=1}^{m!}p\left(\pi_{j}\right)log\left(p\left(\pi_{j}\right)\right).$$
  Here, for all the experimental examples, the embedding dimension is in the range $m=\left[4,7\right]$ [50].

### 2.4. Stationary Wavelet Packet Singular Value Entropy

The stationary wavelet packet singular value entropy (SWPSVE) of the wavelet coefficients matrix *W* is calculated as follows:

(12a) $$\begin{array}{cc} SWPSVE(W) & =-\frac{1}{log_{2}\left(K\right)}\sum_{k=1}^{K}p\left(k\right)log_{2}\left(p\left(k\right)\right)\;K=2^{j} \end{array}$$

(12b) $$\begin{array}{cc} p(k) & =\frac{s_{k}^{2}}{\sum_{k=1}^{K}s_{k}^{2}}, \end{array}$$

where $s_{k}$ corresponds to the *k*-th SV of wavelet packet coefficients matrix *W*, which are obtained using the singular value decomposition method as follows [51]:

(13) $$W=\sum_{k=1}^{K}s_{k}u_{k}v_{k}^{T}=USV^{T},$$

where $U\in R^{K\times K}$, $V\in R^{N\times N}$ represent mutually orthogonal elementary matrices and *S* denotes the K×N diagonal singular values matrix.

## 3. Bearing Fault Diagnosis Algorithm

The algorithm for failure diagnosis presented in this study consists of two phases; the entropy features extraction phase and the classification phase. While the discriminative features extraction phase is carried out by integrating stationary wavelet packet transform and both the dispersion and permutation entropy, the multi-fault classification is performed by means of a KELM model based on the Gaussian kernel function and the *k*-fold cross validation method. We describe this phases in the next sections.

### 3.1. Proposed Diagnosis Algorithm

The steps of the bearing fault diagnosis algorithms proposed in this paper are as follows:

*Step* *1:* Divide the discrete time raw vibration signal into multiple non-overlapped signals of *N* data points.
*Step* *2:* Decompose the non-overlapping signals x(n),n=1,…,N into $D=2^{J}$ sub-band signals by using SWPT given as Equations (1) and (2).
*Step* *3:* Create a *D*-dimensional features vector based on multi-scale wavelet Shannon entropy as follows:
  (14) $$u_{k}=\left[1/E_{1},1/E_{2},\cdots ,1/E_{i},\cdots ,1/E_{D}\right],$$
  where $E_{i}$ represents one of the SWPDE/SWPPE/SWPSVE value of the *i*-th wavelet sub-band signal and *k* corresponds to the *k*-th non-overlapping raw vibration signal.
*Step* *4:* Normalize the features matrix *Z* as follows:
  (15) $$z_{i}=\frac{u_{i}-u_{i,min}}{u_{i,max}-u_{i,min}}\;i=1,2,\cdots ,D,$$
  where $z_{i}$ corresponds to the *i*-th column of the feature matrix *Z*, $u_{i,min}$ and $u_{i,max}$ denote the minimum value and maximum value of the $z_{i}$ vector, respectively.
*Step* *5:* Create the KELM classifier based on both the feature matrix *Z* and *k*-fold cross-validation method.

### 3.2. Kernel-ELM Classifier

In this section we present a brief description of KELM and its main characteristics, based on our previous work on ELM [39] and KELM [40]. For more details on this topic see [36,37,38,52].

The KELM classifier output is obtained as follows:

(16a) $$\begin{array}{cc} \hat{Y}\left(z\right) & =\left(\begin{array}{c} \ker(\tilde{z},z_{1})\\ \ker(\tilde{z},z_{2})\\ \vdots \\ \ker(\tilde{z},z_{M2}) \end{array}\right)\beta \end{array}$$

(16b) $$\begin{array}{cc} \beta & ={\left(\frac{I_{M1}}{C}+Ker\left(\tilde{z},\tilde{z}\right)\right)}^{†}Y, \end{array}$$

where $\tilde{z}\in R^{D\times M1}$ represent the set of input vectors to train, $z\in R^{D\times M2}$ denotes the set of input vectors to test, M1 and M2 represent the samples number of training and testing, respectively. The function ker(·) denotes the Gaussian kernel given as:

(17) $$\ker\left(\tilde{z_{i}},\tilde{z_{j}}\right)=exp\left(-\frac{{∥\tilde{z_{i}}-\tilde{z_{j}}∥}^{2}}{2\sigma^{2}}\right),$$

where the σ parameter corresponds to the kernel width and the σ parameter is set to $\sigma^{2}=log_{10}\left(D\right)$, the *D* value corresponds to the dimensionality of the input features vector to the KELM classifier (see Equation (14)). The $I_{M1}$ is the identity matrix, the β values are output weights of the KELM classifier and the *C* parameter corresponds to the regularisation value. The ${\left(\cdot \right)}^{†}$ expression corresponds to the Moore–Penrose generalized inverse matrix [53] and *Y* corresponds to the desired output pattern matrix.

Finally, the class label predicted for sample *z* is computed as follows:

(18) $$Label\left(\hat{y}\left(z\right)\right)=max\left\{{\hat{y}}_{1}\left(z\right),\ldots ,{\hat{y}}_{10}\left(z\right),{\hat{y}}_{11}\left(z\right),{\hat{y}}_{12}\left(z\right)\right\}.$$

Using the 5-Fold Cross-Validation (CV) method [54,55], the regularisation parameter *C* is chosen from the range $\left\{10^{1},\ldots ,10^{6}\right\}$.

### 3.3. Experimental Setup

**Figure 1:**
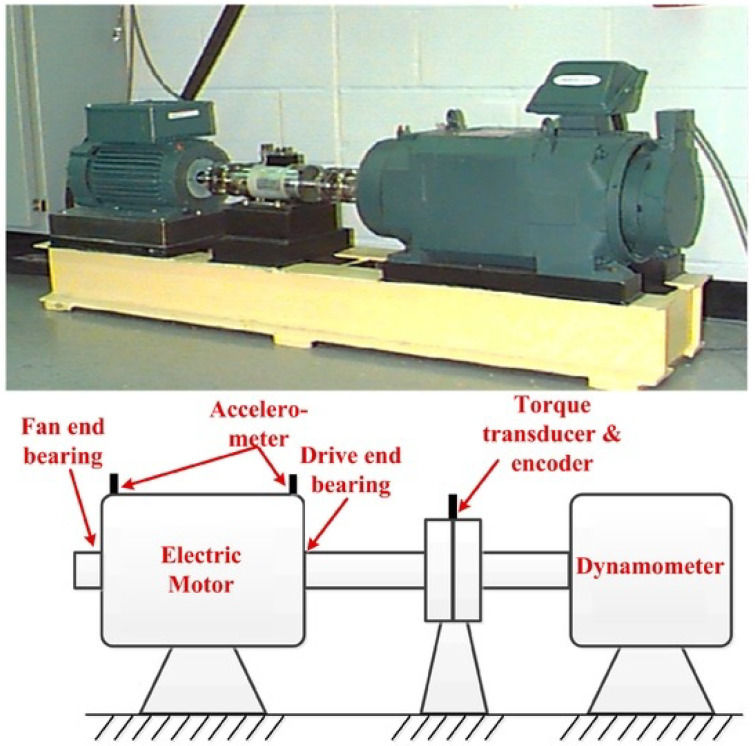
Experimental Setup [56].

In this paper we considered experimental raw data obtained from vibration signals coming from two bearings; the drive-end (6205-2RS JEM SKF, deep groove ball bearing) and the fan-end (6203-2RS JEM SKF, deep groove ball bearing) bearings. These two datasets were obtained from [46]. An experimental setup as the one shown in Figure 1 was used to generate this dataset. This setup consisted of a 2 hp Reliance electric motor, a dynamometer and a torque transducer/encoder. The bearing held the motor shaft during the experiments. In order to collect vibration signals, an accelerometer mounted on the motor housing, as the one shown in Figure 1, was used. Single point failures with different failure diameters of 7, 14, 21 and 28 mils (1 mils =0.001 inch) were introduced to both the driving-end and the fan-end bearings using the electro-discharge machining method, with the motor speed varied at 1730 r/min, 1750 r/min, 1772 r/min, and 1797 r/min for loads of 3, 2, 1, and 0 hp, respectively. Digital data was produced at 12,000 samples per second during 10 seconds for normal bearing (NB) condition samples and failure condition samples; outer race fault (ORF), inner race fault (IRF), and ball fault (BF). Further details on the experimental setup can be found in [46].

## 4. Experimental Results

We performed experiments on the two datasets presented above. With these experiments, we aimed to evaluate the performance of the diagnostic methods proposed in this paper. Firstly, we applied the *J*-levels SWP transform to decompose the non-overlap signal into $D=2^{J}$ sub-band signals. Secondly, the Shannon entropy value was computed using the corresponding Equations (7), (11) or (12) for each wavelet sub-band raw signal. Thirdly, KELM model was applied to diagnose the bearing fault types with different severities. Equations (13) and (15) were used to compute the output weights of the KELM classifier, and a 5-fold cross validation method for each value of *J* was used to adjust the regularization Parameter *C*. The bearing vibration signal database was split into five folds. In order to adjust the parameters *J* and *C*, four out of the five folds were used. The remaining fold was used for the testing stage. To evaluate the performance of the KELM model during the testing stage we considered the following measures of performance:

(19) $$\mathrm{Accuracy}=\frac{1}{M_{2}}\sum_{j=1}^{12}CM_{j,j},$$

where the $M_{2}$ value corresponds to the number of testing samples for all classes combined, CM represents the confusion matrix and $CM_{j,j}$ corresponds to the number of samples in class $y_{j}$ that are correctly classified as class $y_{j}$ [57,58]. The second measure called F-scores was computed for every class label and it is calculated as follows:

(20a) $$\begin{array}{cc} F-\mathrm{scores}(j) & =2\times \frac{\mathrm{Precision}(j)\times \mathrm{Recall}(j)}{\mathrm{Precision}(j)+\mathrm{Recall}(j)}\;j=1,2,\ldots ,10,11,12 \end{array}$$

(20b) $$\begin{array}{cc} \mathrm{Precision}(j) & =\frac{CM_{j,j}}{\sum_{i=1}^{12}CM_{j,i}} \end{array}$$

(20c) $$\begin{array}{cc} \mathrm{Recall}(j) & =\frac{CM_{j,j}}{\sum_{i=1}^{12}CM_{i,j}}, \end{array}$$

where the precision, recall and F-score measures of the *j*-th predicted class are represented by Precision(j), Recall(j), and F-scores(j), respectively [57,58].

### 4.1. Case 1: Drive-End Bearing

The collected dataset considered one normal bearing (NB) condition and 11 faulty bearing conditions that represented all possible combinations of the three possible failure locations (ORF, IRF and BF) over the four different fault severity levels (7, 14, 21 and 28 mils), giving a 12-class identification problem. For each class, there were four vibration signals corresponding to the rotatory shaft speeds of 1797 r/min, 1772 r/min, 1750 r/min and 1730 r/min with loads of 0, 1, 2 and 3 hp, respectively, leaving a total of 48 vibration signals. The length of these raw vibration signal was set to 120,000 data points (obtained in 10 s). Each of these 48 signals was divided into 60 segments. The size of each segment was set to 2000 data points (≈five times the rotation shaft period). Table 1 shows these values.

**Figure 2:**
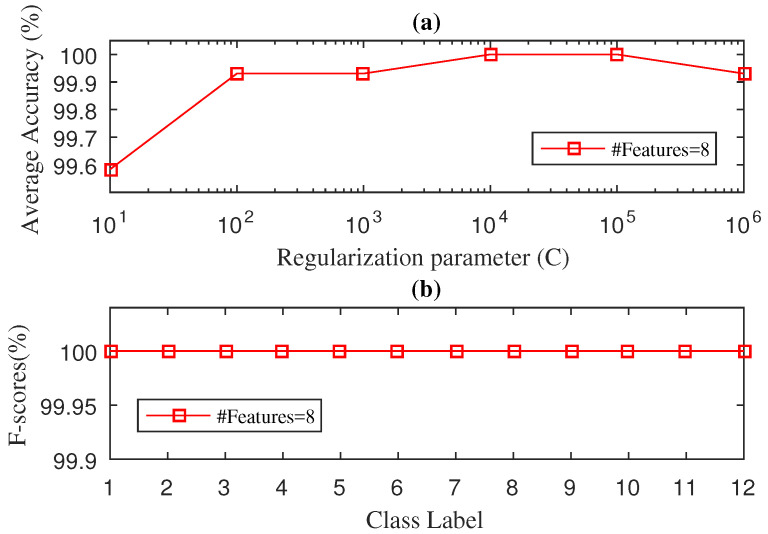
Diagnosis Accuracy (**a**) and F-score (**b**) values obtained by the SWPDE-KELM diagnosis with 5-Fold-CV during testing phase for drive end bearing.

We used the 5-fold cross validation method to find the regularisation parameter *C* and the number of discriminative features. Figure 2 illustrates the average accuracy values and F-score values of the proposed SWPDE–KELM method during the testing stage considering *c* (number of states of the dispersion entropy) equal to 5. The effect on the diagnosis accuracy of other values of *c* can be seen in Table 2. As we can see from Figure 2a, an average accuracy of 100% is achieved considering eight features and the regularization parameter set to $C=10^{4}$, whereas Figure 2b shows the F-score results achieved for twelve different types of faults. As can be seen, there were no misclassified testing samples and the F-score value was 100% for each of the twelve classes, which validated the effectiveness of the proposed SWPDE–KELM method.

**Figure 3:**
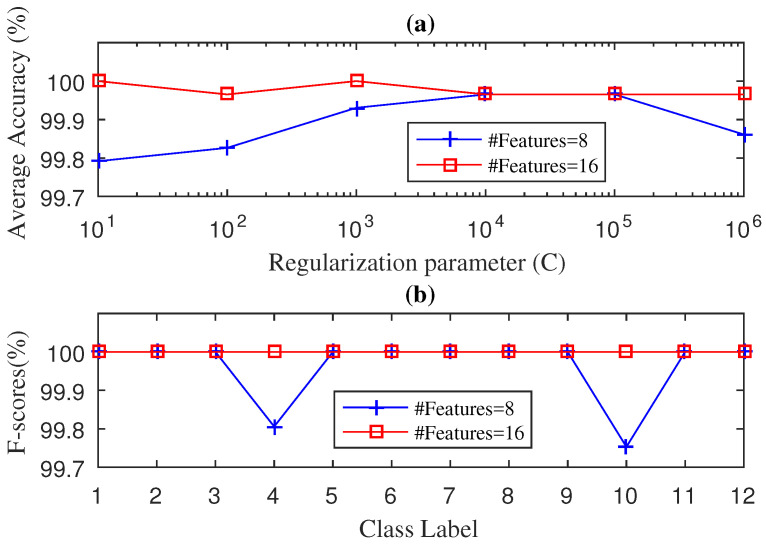
Diagnosis Accuracy (**a**) and F-score (**b**) values obtained by the SWPPE-KELM diagnosis with 5-Fold-CV during testing phase for drive end bearing.

We then tried our SWPPE–KELM method on the drive-end bearing signals. We adjusted both the number of features and the *C* parameter using the same 5-fold cross validation procedure. Figure 3 shows the performance evaluation during the testing phase for the average accuracy values and the F-score values for the SWPPE–KELM method using the embedding dimension m=6. The effect on the diagnosis accuracy of other values of *m* can be seen in Table 2. We can see in Figure 3a, that the SWPPE–KELM method reached an average accuracy of 100% with sixteen features (i.e., 4-level wavelet decomposition) and the regularization parameter set to C=10, while the average accuracy of the SWPPE–KELM method constructed with eight features decreased, and the best average accuracy achieved with eight features was 99.97% for a regularization parameter set equal to $C=10^{4}$. The F-score results for the SWPPE–KELM method with eight and sixteen features are shown in Figure 3b. As can be seen, there were no misclassified test samples when the SWPPE–KELM method was constructed with sixteen features. On the contrary, the method built with eight features achieved an F-score of 100% in only ten classes, since for class 4 (ORF-21 mils) and class 10 (BF-14 mils) it reached an F-score of 99.91% and 99.751%, respectively. Therefore, even though the SWPPE–KELM method achieved an average accuracy of 100%, it needed eight features more than the SWPDE–KELM method, making the SWPDE–KELM slightly more efficient than the SWPPE–KELM method.

**Figure 4:**
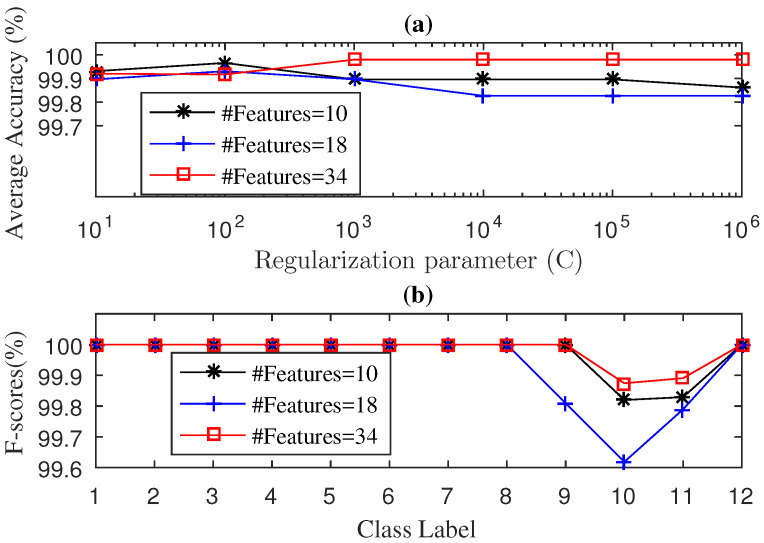
Diagnosis Accuracy (**a**) and F-score (**b**) values obtained by the SWPSVE-KELM diagnosis with 5-Fold-CV during testing phase for drive end bearing.

The average accuracy of the SWPSVE–KELM method during testing phase with $2^{J}+2$ features with $J=\left\{3,4,5\right\}$, where the two extra features corresponded to the Shannon entropy of the raw signal and the Shannon entropy of the singular values, are illustrated in Figure 4a. As we can see in Figure 4a, an average accuracy of 99.98% was achieved for 34 features and the parameter $C=10^{3}$. On the contrary, the average accuracy for 10 and 18 features was generally lower than for 34 features. In Figure 4b we present the F-score results obtained during the testing phase. Overall, the best results were obtained with 34 features. It obtains an F-score of 100% for 10 classes, whereas for class 10 (BF-14 mils) and class 11 (BF-21 mils), the F-score achieved was of 99.87% and 99.89%, respectively. In addition, from Figure 4b it can be observed that the F-score results worsen with 10 and 18 features.

Therefore, from these results, diagnosis accuracy obtained by the SWPDE–KELM method was slightly better than the SWPPE–KELM method and they both significantly outperformed the SWPSVE–KELM method when applied to the drive-end dataset.

### 4.2. Case 2: Fan-End Bearing

For the fan-end bearing dataset we used in this paper, collected data consisted of nine faulty bearing conditions with three failure diameters (7, 14 and 21 mils) and a normal bearing condition, giving a 10-class recognition problem. For each class, there are 240 samples and a total of 2400 samples. We use the same 5-fold cross validation method to find parameters *C* and *J*.

**Figure 5:**
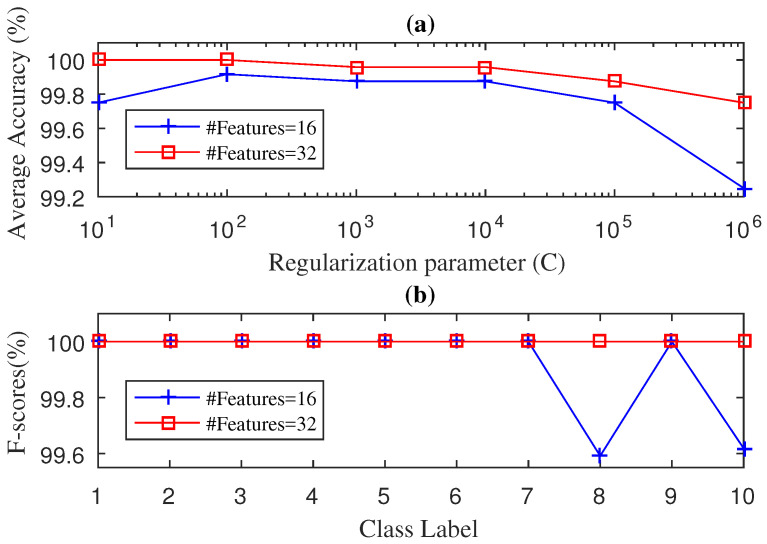
Diagnosis Accuracy (**a**) and F-score (**b**) values obtained by the SWPDE-KELM diagnosis with 5-Fold-CV during testing phase for fan-end bearing.

Figure 5 shows the average accuracy and the F-score values obtained by the SWPDE–KELM method considering *c* (number of states of the dispertion entropy) equal to 5. The effect on the diagnosis accuracy of other values of *c* can be seen in Table 3. As we can see in Figure 5a, when 16 features are considered (J=4), the method cannot reach 100% average accuracy. If we increase the number of features to 32 (J=5), the 100% of average accuracy is only reached for $C=\{10^{1},10^{2}\}$ values. It was interesting to note that for both values of *J*, as the parameter *C* increased ($C>10^{2}$), the average accuracy was heavily impaired. We then compute the F-score values using 16 and 32 features with $C=10^{2}$ and $C=10^{1}$, respectively. As we can see in Figure 5b, when we used 32 features the SWPDE–KELM method reached the 100% F-score value for the all 10 failure classes. However, when we used 16 features, the method reached the the 100% F-score value for only eight out of the 10 failure classes. It was also interesting to note that the two failure classes for which our method was not able to reach the 100% F-score value corresponded to ball faults (7 mils and 21 mils). This might mean that ball faults are more difficult to identify. We can also note that failures in this dataset (fan-end bearing) were harder to identify than the ones in the drive-end bearing dataset.

**Figure 6:**
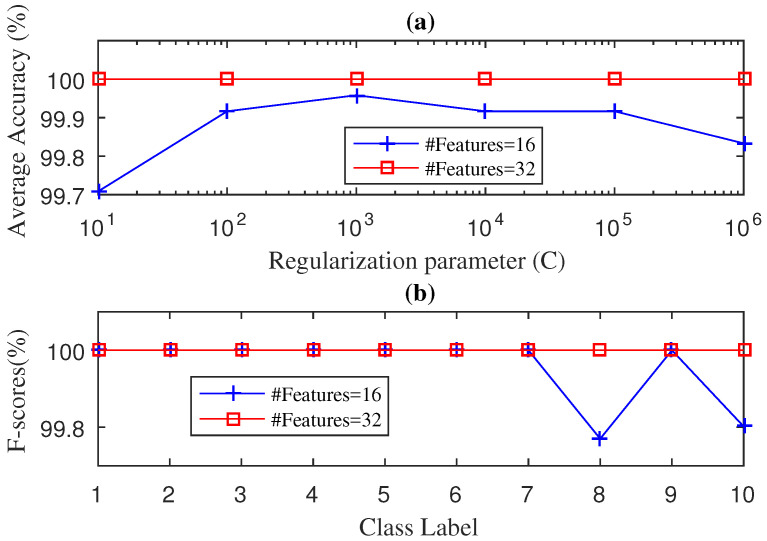
Diagnosis Accuracy (**a**) and F-score (**b**) values obtained by the SWPPE-KELM diagnosis with 5-Fold-CV during testing phase for fan-end bearing.

Figure 6 shows the average accuracy and the F-score values obtained by the SWPPE–KELM method using the embedding dimension m=6. The effect on the diagnosis accuracy of other values of *m* can be seen in Table 3. Just as for the SWPDE–KELM method, when 16 features are considered (J=4), the method cannot reach the 100% of average accuracy and, again, if we increase the number of features to 32 (J=5), the 100% of average accuracy is reached for all values of *C*. We then computed the F-score values using 16 and 32 features with $C=10^{3}$ and $C=10^{1}$, respectively. As we can see in Figure 6b, when we used 32 features the SWPPE–KELM method reached the 100% F-score values for the all 10 failure classes. Again, when we used 16 features, the method reached the the 100% F-score values for only eight out of the 10 failure classes. Ball faults were, again, the only two failure classes for which our method was not able to reach the 100% F-score value. It is important to note that, although the number of times both the SWPDE–KELM and the SWPPE–KELM methods reach the 100% F-score value was the same, the SWPPE–KELM method was slightly better than the the SWPDE–KELM method for those fault classes for which the 100% F-score value was not reached.

**Figure 7:**
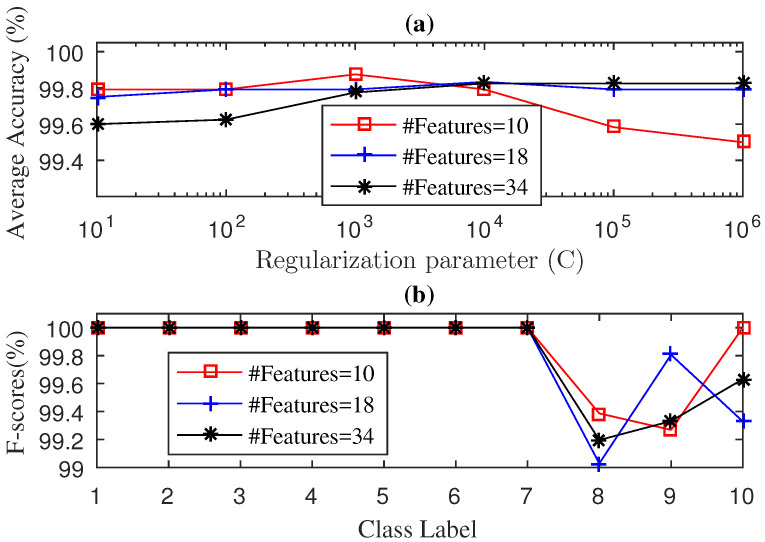
Diagnosis Accuracy (**a**) and F-score (**b**) values obtained by the SWPSVE-KELM diagnosis with 5-Fold-CV during testing phase for fan-end bearing.

Finally, in Figure 7 we present the results obtained by the SWPSVE–KELM algorithm introduced in [40]. For this method we considered, again, $2^{J}+2$ features with J={3,4,5}. Figure 7a shows the average accuracy for different values of *C* and *J*. Unlike the methods proposed here, the SWPSVE–KELM method never reached the 100% of average accuracy. The best average accuracy value (99.88%) was obtained for $C=10^{3}$ and 10 features. For 18 and 34 features, the best average accuracy value (99.83%) was obtained when $C=10^{4}$. Once we set values of *C* for each value of *J* we computed the F-score. As we can see in Figure 7b, when we used 10 features, the SWPSVE–KELM method reached the 100% F-score value in eight out of the 10 failure classes. When we used 18 and 34 features, the 100% F-score value was only reached in seven out of the 10 failure classes. We need to point out that, just as for our methods, ball faults are much harder to identify, however, results obtained by our methods clearly outperformed the ones obtained by the SWPSVE–KELM method.

## 5. Conclusions

This article presents two methods, called SWPDE and SWPPE, for feature extraction for bearing failure diagnosis. Our proposed methods combine SWP transform and Shannon entropy to improve the accuracy of the classifier. More specifically, the probability distribution function of the entropy is computed using either differential or permutation entropy.

Drive-end and a fan-end bearing datasets are considered in this study. We apply our algorithms on these datasets and compare our results to those obtained by a recently reported method called SWPSVE–KELM.

For the drive-end bearing dataset, we found that our SWPDE–KELM method reached a 100% accuracy level and 100% F-score value for the 12 bearing work conditions using 8 features. Although the SWPPE–KELM method also reached a 100% accuracy level and 100% F-score value, it needed 16 features to do so. These values are still very good when compared to the 34 features that the SWPSVE–KELM needs to reach similar values.

For the fan-end bearing dataset, our proposed methods reach the 100% F-score value for the all 10 failure classes. Since failures in the fan-end dataset are much harder to identify, our methods need 32 features to reach the 100% F-score value. Although the SWPSVE–KELM method only reached the 100% F-score value in eight out of the 10 failure classes, it only needed 10 features.

It is also interesting to note that, when considering only 16 features, our method was not able to reach the 100% F-score value for two classes corresponding to ball faults (7 mils and 21 mils). This might mean that ball faults are more difficult to identify. Furthermore, these failures in the fan-end bearing are harder to identify than the ones in the drive-end bearing dataset. However, although ball faults are much harder to identify, results obtained by our methods for these classes of failures are much better than the ones obtained by the SWPSVE–KELM method.

Although only experimental data has been considered in our experiments, it is important to note that, as has been shown in the literature previously, diagnosis algorithms tuned using experimental data can be succesfully used in more realistic environments for fan-end bearings [14].

Overall, our methods clearly outperform the SWPSVE–KELM method w.r.t. F-score and accuracy. Further, as shown in Table 4, our method is able to reach the same diagnosis accuracy levels as other previously proposed methods with a lower complexity of parameter tuning. As future work, we aim to combine Shannon entropy and different time–frequency analysis methods to rotationary machine failure diagnosis under variable work conditions. Also, we intend to apply our algorithms to more realistic datasets such as the ones from planetary bearings and gearboxes.

## Figures and Tables

**Table 1:** Structure of bearing datasets.

Fault Types	Speed (r/min)	Load (hp)	Fault Diameter (mils)	Samples Numbers	Class Label ${}^{1}$	Class Label ${}^{2}$
NB	1797–1730	0–3	0	240	1	1
ORF	1797–1730	0–3	7	240	2	2
			14	240	3	3
			21	240	4	4
IRF	1797–1730	0–3	7	240	5	5
			14	240	6	6
			21	240	7	7
			28	240	8	–
BF	1797–1730	0–3	7	240	9	8
			14	240	10	9
			21	240	11	10
			28	240	12	–

${}^{1}$ drive-end bearing; ${}^{2}$ fan-end bearing.

**Table 2:** Entropy’s parameter for drive-end bearing.

Method	Embedding (*m*)	Classes (*c*)	Avg. Accuracy
3-level SWPDE	2	5	100
		6	100
		7	100
		8	100
4-level SWPPE	4	——	99.97
	5		99.97
	6		100
	7		100

**Table 3:** Entropy’s parameter for Fan-end Bearing.

Method	Embedding (*m*)	Clases (*c*)	Avg. Accuracy
3-level SWPDE	2	5	100
		6	100
		7	100
		8	100
4-level SWPPE	4	——	99.93
	5		99.97
	6		100
	7		100

**Table 4:** Comparison between the proposed method and some previous work for bearing fault diagnosis.

Reference	Feature Extraction	Classification Method	Classes Number	Average Accuracy (%)
Brkovic et al. [14]	Wavelet energy entropy	Quadratic Classifier	4	100
Li et al. [59]	MPE from LMD	SVM with Binary Tree	4	100
Zheng et al. [60]	FE from LCD	ANFIS	7	100
Yan et al. [61]	IED-PE from IVMD	KNN	8	98.38
[40]	Singular entropy from stationary wavelet	KELM	10	100
Mao et al. [62]	Fourier amplitude	Deep-ELM	10	100
Yan and Jia [63]	Multi-domain features with Laplace score	SVM with PSO	12	100
This work	DE and PE from stationary wavelet	KELM	12	100
